# Arthroscopic management of elbow stiffness

**DOI:** 10.1186/s40634-021-00420-4

**Published:** 2021-10-28

**Authors:** Joaquin Sanchez-Sotelo

**Affiliations:** Chair of the Division of Shoulder and Elbow Surgery, Department of Orthopedic Surgery, Mayo Clinic, Gonda 14, 200 First Street SW, MN 55905 Rochester, USA

## Abstract

The elbow is particularly prone to stiffness. Loss of elbow motion is very limiting, and can be the result of trauma, primary osteoarthritis, heterotopic ossification and other conditions. Several exposures have been described for open elbow contracture release. Although a few decades ago elbow arthroscopy was considered only for diagnosis and removal of loose bodies, contemporary arthroscopic techniques allow successful management of the majority of conditions leading to elbow stiffness. Careful patient evaluation, use of advanced imaging studies, and acquisition of appropriate surgical skills are essential for the successful arthroscopic management of the stiff elbow. This expert opinion reviews some fundamentals of elbow stiffness as well as principles for the evaluation and arthroscopic management of the stiff elbow.

## Introduction

Loss of elbow motion is a relatively common complication of elbow trauma, and it can also occur as a consequence of primary elbow osteoarthritis, as well as inflammatory and other conditions [11, 19]. Most individuals experience substantial functional difficulties when loss of elbow motion exceeds certain thresholds: activities such as shaving and buttoning a shirt become difficult with loss of flexion, whereas loss of elbow extension makes it difficult to reach out. High-performance sports may become impossible without restoration of normal motion; for example, gymnastics and ballet require complete terminal extension [5].

Whereas conservative treatment may improve motion to some degree in patients recovering from a traumatic injury, oftentimes elbow motion can only be improved with surgery. Fortunately, arthroscopic elbow techniques have been refined to allow successful surgical management of the majority of stiff elbows. This expert opinion review summarizes our current understanding of elbow stiffness and some of the principles for successful arthroscopic management of the stiff elbow.

### Understanding elbow stiffness

For orthopedic surgeons, elbow stiffness implies that the elbow joint has lost motion in one or more directions (flexion, extension, pronation and/or supination). Several factors may contribute to loss of motion, including fibrosis, osteophytes, ectopic bone formation, and changes to the joint articular surface.

#### Arthrofibrosis, neurogenic contracture and heterotopic ossification

Excessive capsular fibrosis (*arthrofibrosis*) may be the only pathologic change contributing to motion loss in certain elbows, but it certainly contributes to motion loss for most stiff elbows secondary to any of the other mechanisms discussed in this review. The capsule in arthrofibrosis is (a) thicker, (2) less elastic, and (3) adheres abnormally to the margins of the joint (or even portions of the articular surface). Investigations from our laboratory seem to indicate that excessive myofibroblastic activity is the main cellular mechanism of arthrofibrosis [1]. A complex interaction between immune-inflammatory cells, mast cells, macrophages, stem cells and fibroblasts seem to result in preferential differentiation to fibrotic as opposed to adipose tissue with myofibroblasts that over-proliferate, produce excessive fibrotic matrix, and lose the ability to undergo programmed cell death (apoptosis) [3, 8].

Although inflammatory mediators are predominantly involved in the genesis of most arthrofibrotic elbows, we have identified patients in our practice where the only inciting mechanism seem to be neuritis, involving either the ulnar nerve or cutaneous nerves. These *neurogenic contractures* may resolve when the neuritis is addressed, but oftentimes present with secondary arthrofibrosis as well. Conceptually, elbow neurogenic contractures can be considered part of the spectrum of complex regional pain syndrome. Neurogenic modulators such as substance P may be involved.

The elbow is also well known for its propensity to develop formation of *ectopic bone* after elbow trauma, elbow surgery, and in certain individuals after an injury to the central nervous system (brain or spinal cord injuries) or extensive burns [25]. When loss of elbow motion is attributed to heterotopic ossification, there is almost always arthrofibrosis as well. In addition, release of nerve mediators may be associated with the cascade that triggers ectopic bone formation.

#### Osteophytes and the articular surface

Elbow stiffness may also be secondary to abnormalities of the articular surface or osteophyte formation. *Malunion* after trauma may lead to incongruence that limits motion. Articular cartilage degeneration may also contribute to loss of motion. Stiffness after elbow trauma may be secondary to arthrofibrosis in isolation (for example, stiffness after a simple elbow dislocation), but more often it is an expression of *posttraumatic arthritis,* combining arthrofibrosis with a painful, abnormal articular surface. *Primary elbow osteoarthritis* is very different: loss of motion is mostly secondary to mechanical impingement of loose bodies and osteophytes, with arthrofibrosis contributing, but to a lesser extent [19]. The term *extrinsic contracture* refers to those circumstances when the articular surface does not need to be addressed to restore motion, whereas motion in elbows with *intrinsic contracture* cannot be restored reliably without addressing the articular surface (i.e., elbow arthroplasty, interposition arthroplasty, osteotomy or resection of the articulation would be required to restore motion).

#### The ulnar nerve

In addition to contributing to selected cases of neurogenic contracture, the ulnar nerve may also become damaged by a sudden increase in motion with surgery. In the stiff elbow, the ulnar nerve may be encased by the fibrotic process and/or compressed by osteophytes or other posttraumatic bone irregularity; in addition, nerve excursion has been limited for some time [32]. If at the time of surgery, the ulnar nerve is not decompressed or transposed, it may be at risk for ulnar neuropathy during the first few days to weeks of the procedure (delayed-onset ulnar neuropathy [4]), or at a minimum be painful enough to impede restoration of flexion.

In our practice, we have migrated towards decompressing the ulnar nerve surgically in all elbows that undergo arthroscopic surgery to restore motion. For those surgeons not performing ulnar nerve decompression universally, most agree the ulnar nerve should be addressed surgically in elbows with (1) positive preoperative ulnar nerve abnormalities (sensory and/or motor), (2) flexion less than 90 degrees, (3) radiographic or CT scan evidence of osteophyte or ectopic bone formation that could impinge on the ulnar nerve, and/or (4) a subluxing ulnar nerve with or without a snapping triceps [6].

#### Forearm rotation contractures

Restoration of forearm rotation has not been as commonly discussed in the literature as restoration of a functional flexion-extension elbow arc. Forearm rotation may be impaired by abnormalities of the wrist, radius, ulna, interosseous membrane, formation of ectopic bone, or excessive scar tissue development around the radial head and neck. Advanced arthroscopic elbow techniques may be considered when forearm rotation may be improved by releasing scar tissue around the radial head and neck, removing ectopic bone in that location, or performing an arthroscopic radial head resection.

### Patient evaluation

#### History and physical examination

The primary goals of the evaluation of patients presenting with a chief complaint of elbow stiffness include (1) accurate and precise measurement and documentation of motion, (2) determination of motion needs, (3) identification of the etiology and contributing factors, (4) careful assessment of the condition of the ulnar nerve, and (5) review of prior treatment attempts.

**Fig. 1:**
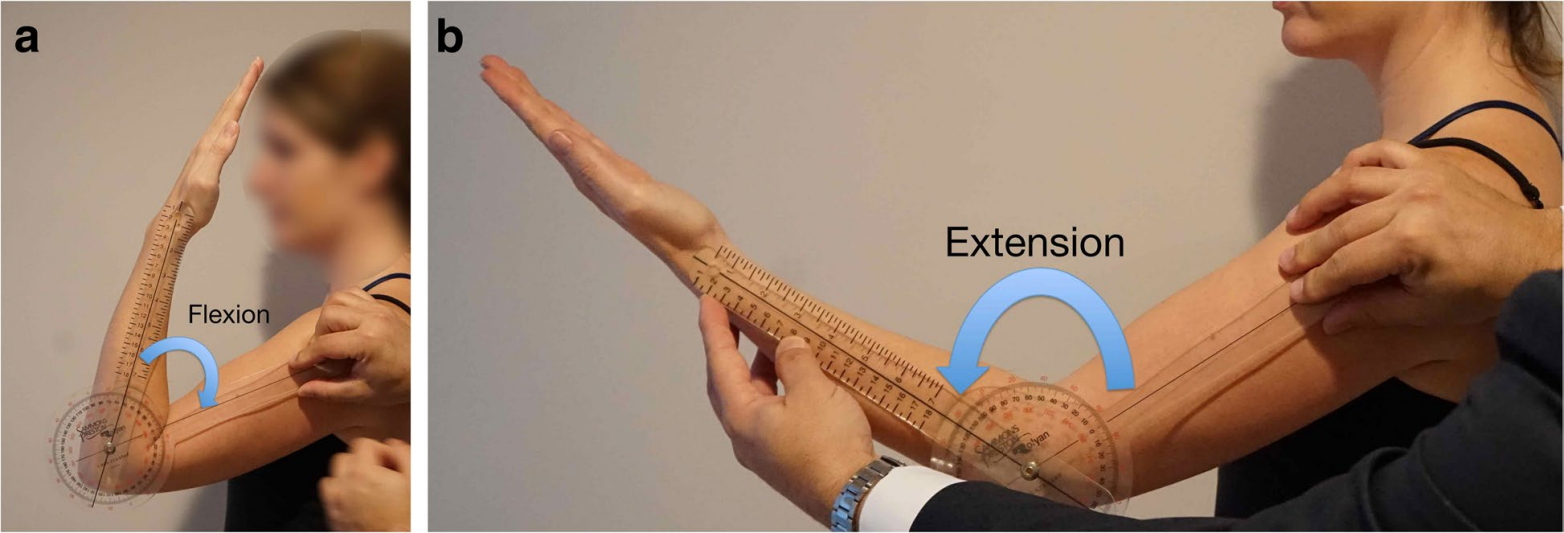
Clinical assessment of elbow flexion (**A**) and extension (**B**) is best performed with the forearm in supination and the flexion-extension axis parallel to the floor using a goniometer

We believe there is a fair amount of variability amongst surgeons when measuring elbow range of motion. As mentioned later, this variability likely has a profound effect on the outcomes reported in the literature when discussing the management of elbow stiffness. As such, we strongly recommend use of a goniometer and careful positioning of the upper extremity. For flexion-extension measurements, the forearm is placed in complete supination if possible and the elbow axis (line connecting both epicondyles) parallel to the floor. For pronation-supination measurements, the elbow should be flexed at 90 degrees with the arm at the side of the chest and forearm rotation may be measured using the extended thumb or an object such as a pen held by the grip of the hand (Fig. 1). Telemedicine has become increasingly common, especially in the light of the CoViD-19 pandemic; dedicated instructions to patients allow accurate and precise measurements of motion remotely.

Once motion has been measured, the recommendation to proceed with surgery will be heavily influenced by *motion needs*. A sedentary patient that seems to be coping well with a flexion-extension arc from − 35 to 125 degrees may not benefit substantially from surgery; on the contrary, a high-performance gymnast may need to be considered for surgery even when loss of extension is relatively minor, in the 15-to-20-degree range [5]. In general, patients with the most severe contractures benefit from surgery the most, but the decision to proceed with surgery should be individualized based on motion needs.

A key element in the evaluation of patients with *loss of motion and pain* is aimed to determine how much the articular surface contributes to symptoms. Pain at rest and pain at night are suggestive of substantial articular cartilage involvement. When pain increases with resisted flexion and extension in the mid-arc (*articular shear test*), the joint surface may need to be addressed in order to improve pain and function. On the contrary, many patients only experience pain on examination when their elbow is suddenly forced into terminal flexion or terminal extension, indicative of *painful impingement* (fractured osteophytes, pinched synovial tissue).

Understanding the reasons for elbow stiffness is critical: is there a history of elbow trauma that required surgery? Does the patient report a history of heavy lifting (construction work, weight-training, farming) typically associated with primary osteoarthritis? Can risk factors be identified in patients presenting with heterotopic ossification? Is there evidence of neuropathic pain or neurologic deficits consistent with neurogenic contracture? What is the condition of the ulnar nerve? Review of imaging and other studies will further clarify all elements contributing to loss of elbow motion. It is important to be aware of the possibility of *refractory elbow arthrofibrosis*, likely an expression of either a very abnormal individual response or an unrecognized neurogenic contracture [21]. It is also important to be aware of rare cases of spontaneous painful stiffness as a result of an *osteoid osteoma* at the elbow; severe pain (especially at night), improvement of pain with non-steroidal anti-inflammatory drugs, and essentially normal radiographs should prompt the possibility of osteoid osteoma.

#### Imaging studies

**Fig. 2:**
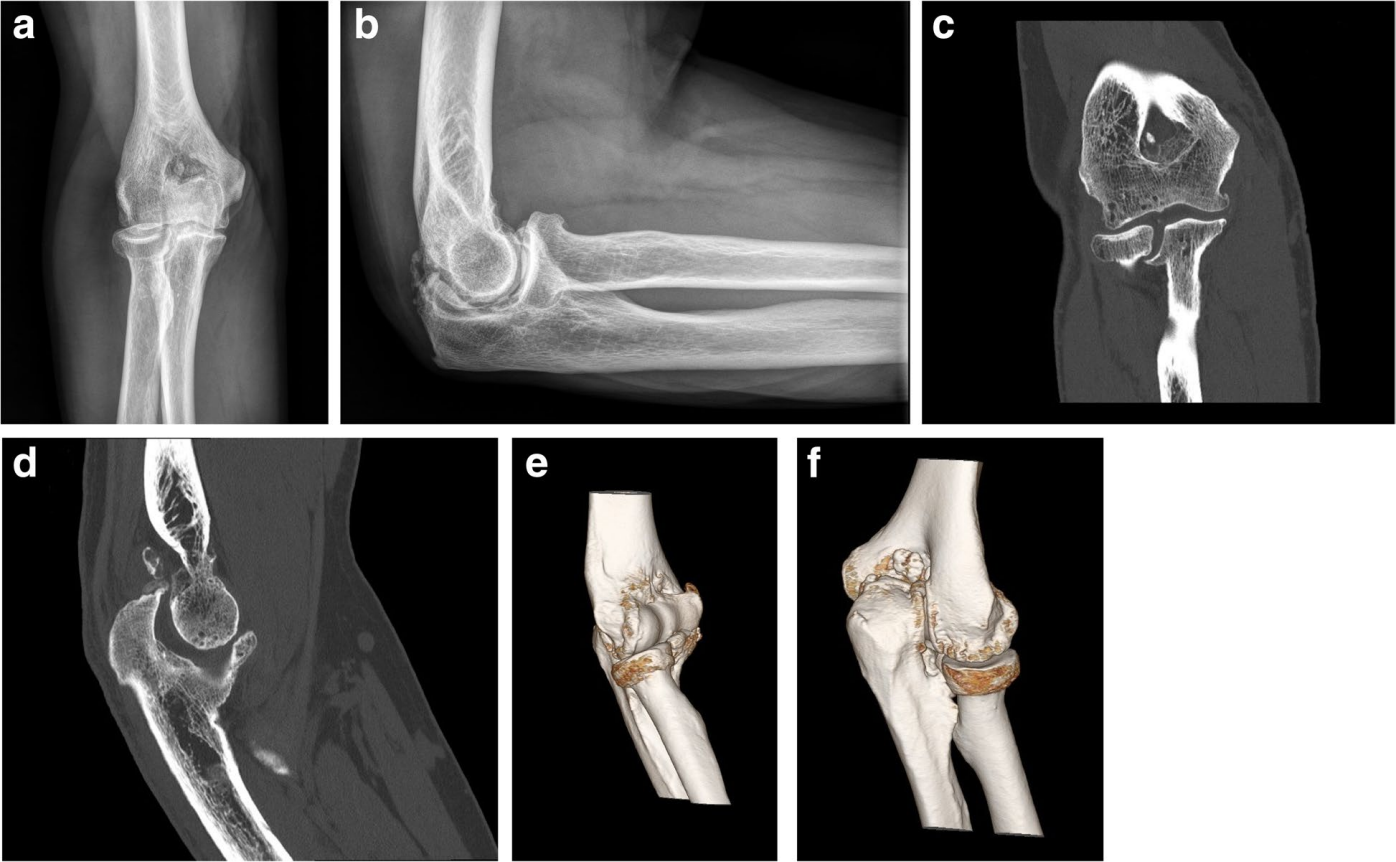
**A** and **B** Anteroposterior and lateral radiographs of an elbow with primary osteoarthritis. **C – F** Computed tomography with three-dimensional rendering provide is extremely useful in the evaluation of stiff elbows

Plain *radiographs* should be obtained in all patients and may be diagnostic of the underlying condition (primary osteoarthritis, posttraumatic osteoarthritis, malunion, heterotopic ossification, osteochondritis dissecans). For patients considered for surgery, *computed tomography* with three-dimensional reconstruction is our imaging modality of choice (Fig. 2); it is very useful for the assessment of osteophyte location, extent and location heterotopic ossification, joint line deformity, osteochondritis dissecans, and even identification of occult osteoid osteomas. In addition, CT scan DICOM files can be segmented and used to *three-dimensionally print* replicas of the skeleton, which can be of value to understand the underlying pathology and even practice certain procedures (ectopic bone removal, osteotomy) when needed. Even though *magnetic resonance* may be considered for assessment of the articular cartilage in arthritic conditions and OCD, we rarely use it. *Ultrasound* is used in our practice to (1) trace the location of the ulnar nerve in patients with a prior transposition, and (2) perform selective diagnostic injections in patients with suspected neurogenic contractures.

#### Electromyogram with nerve conduction studies

These studies may be useful to grade the severity of pre-existing ulnar neuropathy or other nerve deficits. Occasionally, they can be suggestive of chronic regional pain syndrome. It is important to realize that nerve conduction studies are commonly normal in patients with neurogenic contractures secondary to neuropathy of sensory cutaneous nerves.

### Surgical technique

#### Decision making

At the conclusion of the evaluation of patients presenting with a chief complaint of elbow stiffness, three key questions should be answered: a) should surgery be recommended? b) What elements should surgery include? and c) can the procedure be performed arthroscopically?

The answer to the first question will be relatively easy comparing loss of motion with motion needs; in addition, surgery is more commonly recommended when pain is a substantial complaint (in addition to loss of motion).

Regarding which elements should surgery include, most stiff elbows will require removal of the abnormal capsule and bone reshaping, a procedure commonly known as *osteocapsular arthroplasty*; as mentioned previously, we favor routine decompression of the ulnar nerve. Additional procedures needed will depend on the etiology: removal of ectopic bone, removal of an osteoid osteoma, cutaneous neurectomy, hardware removal, radial head removal, surgical management of OCD, and other.

Regarding when to perform the procedure arthroscopically, we believe that osteocapsular arthroplasty should be performed arthroscopically for (1) primary elbow osteoarthritis, (2) most patients with posttraumatic extrinsic stiffness, (3) patients requiring radial head resection, and (4) OCD lesions best suited for retroarticular drilling, debridement or microfracture. Arthroscopic removal of ectopic bone, arthroscopic release of forearm contractures, arthroscopic removal of osteoid osteomas, and arthroscopically assisted hardware removal require unique expertise.

The complexity of pathology that can be treated arthroscopically largely depends on the experience and skills of each surgeon; these procedures are definitely associated with somewhat of a learning curve [13]. It is essential to remember that the neurovascular structures of the upper limb are very close to the elbow capsule, and the risk of nerve injury should not be underestimated [7, 10]. Strategies to prevent nerve injury include (1) a three-dimensional understanding of the anatomy of these nerves, (2) only using bone and tissue removing instruments under direct arthroscopic visualization, (3) avoiding uncontrolled suction through bone and tissue removing instruments, and (4) using arthroscopic retractors, amongst others.

#### Arthroscopic osteocapsular arthroplasty

This procedure involves removing all areas of impinging bone that limit motion and removing the majority of the anterior and posterior capsule.

##### Positioning

We prefer the lateral position, but the procedure may also be performed in the supine or prone position. A tourniquet is applied, and the upper arm is supported on a dedicated arm holder. One key of positioning is to ensure that there will be adequate room for instruments between the elbow and the trunk to work on the anterior compartment; this should be confirmed before draping. Our preference is to address the anterior compartment first because open access to the posterior compartment if needed is associated with less mobility.

##### Technique

**Fig. 3:**
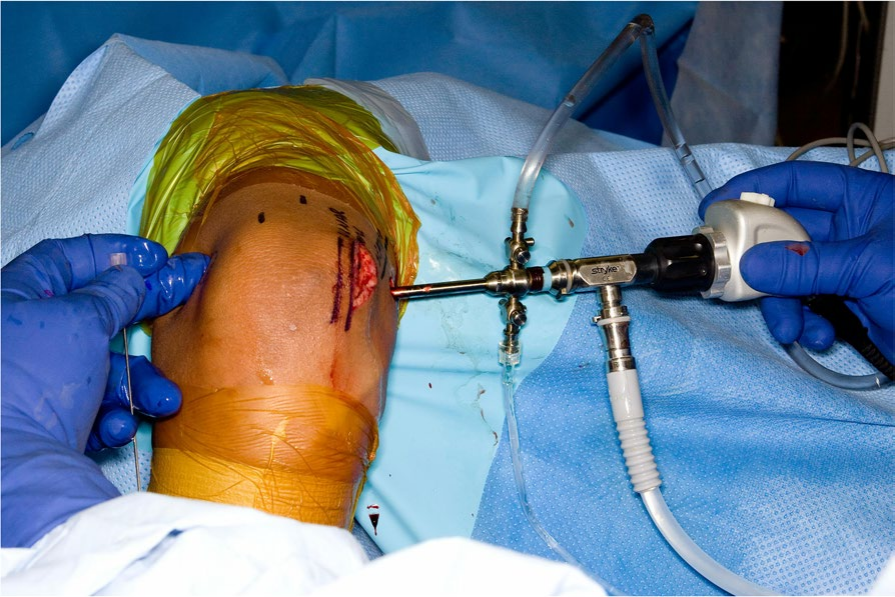
Arthroscopic osteocapsular arthroplasty performed in the lateral decubitus position. In situ decompression of the ulnar nerve through a small incision is performed prior to the arthroscopic portion

In most elbows, the first step of the procedure is to perform an in-situ decompression of the ulnar nerve through a small posteromedial skin incision. By mobilizing skin flaps, it is possible to decompress the nerve 4 cm proximal and 4 cm distal to the medial epicondyle. Gentle medial retraction of the ulnar nerve also allows open resection of the posterior band of the medial collateral ligament open, which is very safe and effective (Fig. 3).

**Fig. 4:**
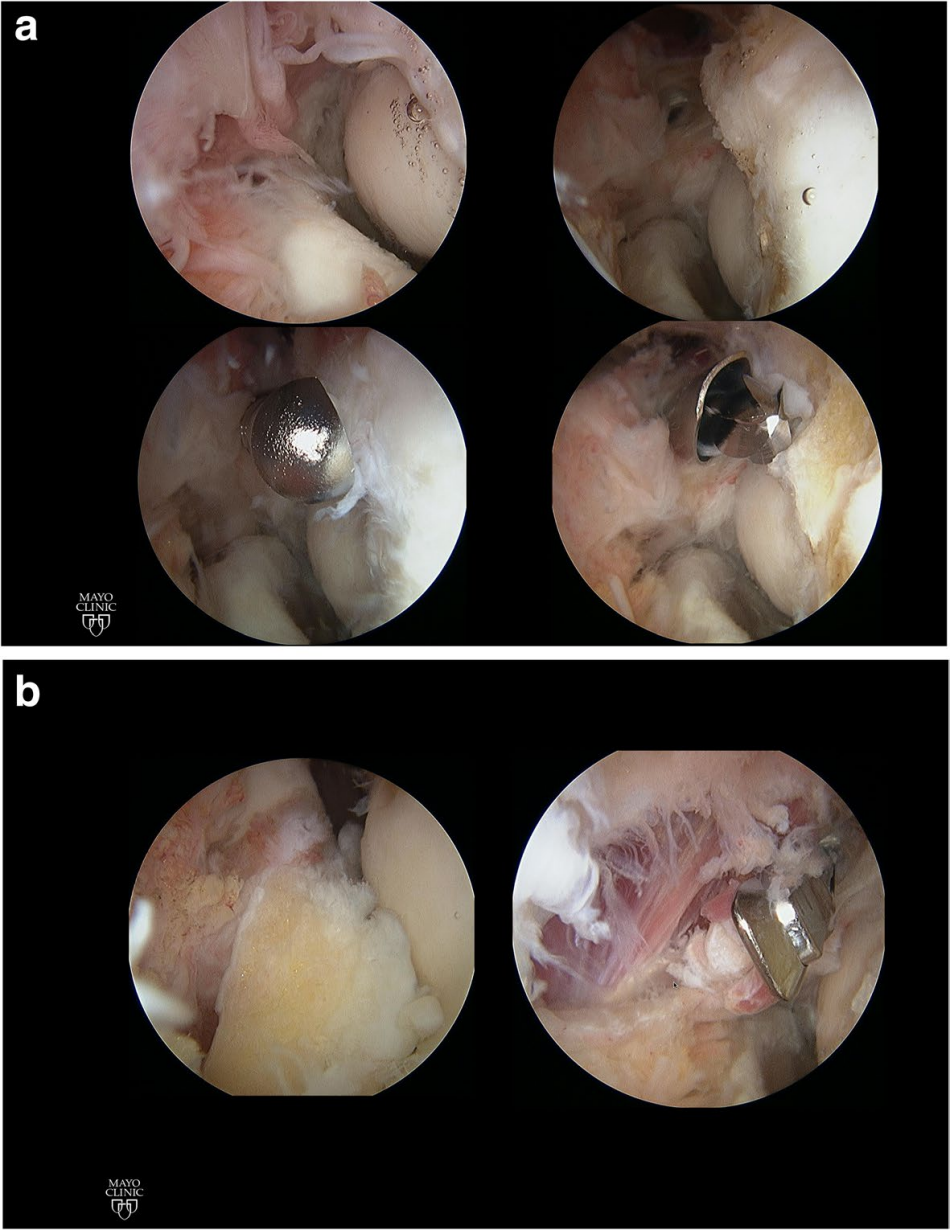
Arthroscopic images show the various steps of elbow osteocapsular arthroplasty. **A** Establishing a view and bone work. **B** Bony work finalized, anterior capsulectomy

The arthroscopic portion of the procedure is performed next. We prefer to address the anterior compartment first. Use of a switching stick to pierce the capsule is a very useful technique to establish portals. The proximal anteromedial portal (anterior and proximal to the medial epicondyle) is established first and used for visualization. The anterolateral portal (anterior and slightly proximal to the capitellum) is established next and used as a working portal. A proximal anterolateral portal is incredibly useful to insert an intraarticular retractor that will displace the capsule and musculature anteriorly. We follow the steps recommended by O’Driscoll [22]: (1) get in and establish a view (2) create a space in which to work, (3) remove bone, and (4) capsulectomy (Fig. 4). Working instruments typically include a shaver, a bur, and a radiofrequency ablation device. The arthroscopic camera and instruments are switched from side to side as needed. An additional anteromedial portal may be useful as well. Care must be taken when removing capsule just anterior to the equator of the radial head, extremely close to the location of the posterior interosseous nerve.

The posterior compartment is addressed in a similar fashion using the posterolateral portal for visualization initially and the mid-central portal for bone and tissue removal. A proximal anterolateral portal may be used for retraction and additional portals, such as one at the soft spot between radial head, olecranon and humerus, are established as needed. Care must be taken when removing bone or tissue posteromedial, in the vicinity of the ulnar nerve.

##### Nuances in posttraumatic osteoarthritis

Typically, access to the articular space is substantially more difficult in posttraumatic than primary osteoarthritis: the capsule is thicker, and it may be adhered to portions of the articular cartilage. Additionally, various degrees of posttraumatic deformity and retained hardware may add complexity [30]. Hardware from the radial head and coronoid can occasionally be removed under arthroscopic visualization, but hardware in other locations (distal humerus, ulna) may require a combined arthroscopic/open approach (or performing the whole procedure open). Care must be taken to avoid manipulation of the elbow after hardware removal to avoid the possibility of an intraoperative fracture through the stress-risers left by screw holes. Removing hardware last, once motion has been regained, helps decrease the risk of intraoperative fracture.

##### Nuances in inflammatory arthritis

Elbows with stiffness secondary to inflammatory arthritis are oftentimes also painful. Synovitis may make arthroscopic visualization of the joint difficult. In addition, the capsule is sometimes thinner than expected [28]. Those two factors combined likely increase the risk of iatrogenic injury to the posterior interosseous nerve. Arthroscopic management of the stiff inflammatory elbow oftentimes also required synovectomy around the radial neck, and occasionally arthroscopic radial head resection. For patients on disease-modifying antirheumatic drugs, the pharmacokinetic properties of each agent need to be reviewed to understand when and for how long to discontinue them around the time of surgery in order to decrease the risk of infection. Inflammatory stiff elbows typically respond well to arthroscopic synovectomy and capsular release even in the presence of moderate radiographic arthritic changes.

##### Prior ulnar nerve transposition

**Fig. 5:**
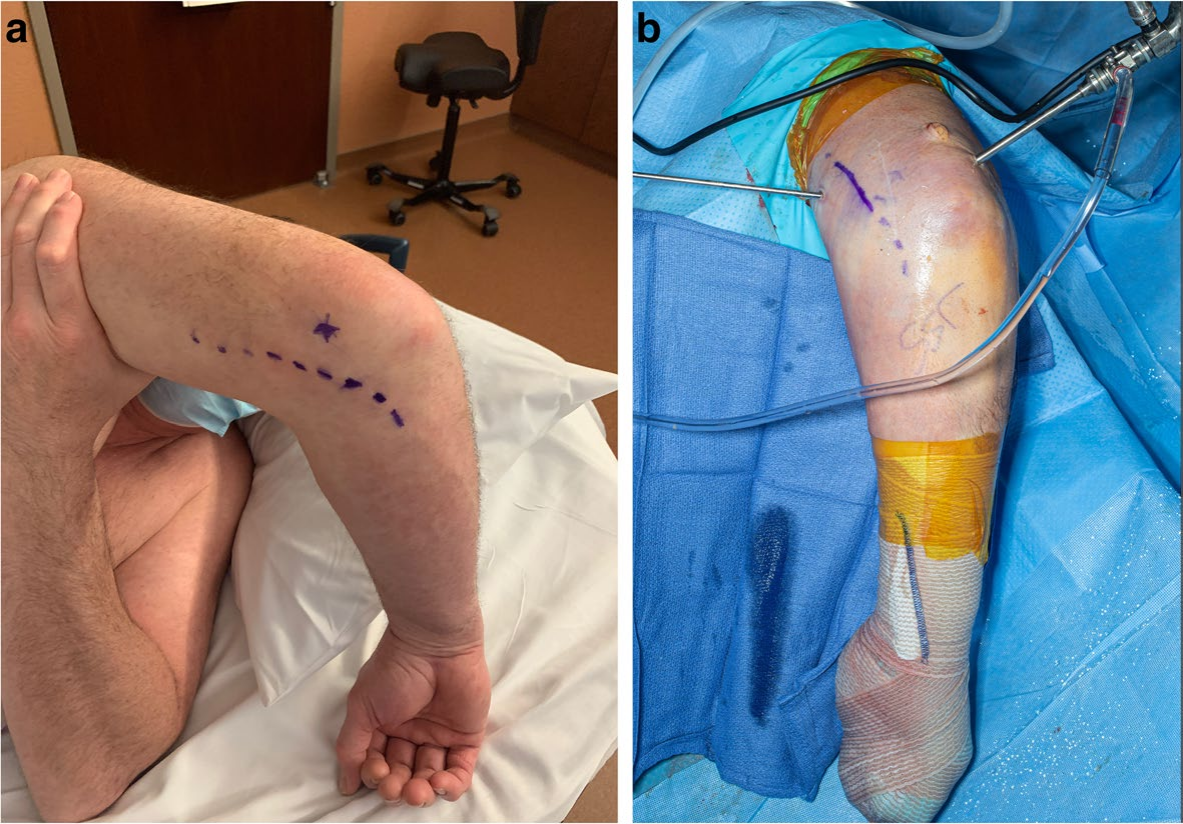
Ultrasound tracing of the course of a transposed ulnar nerve may allow safe arthroscopic osteocapsular arthroplasty. **A** Marking of the ulnar nerve course prior to arthroscopy. **B** Arthroscopic portals avoiding the location of the previously transposed ulnar nerve

Patients presenting with elbow stiffness may have undergone an ulnar nerve transposition previously. Anterior and medial portals risk injuring the ulnar nerve, and as such many consider this a contraindication for arthroscopic contracture release. However, preoperative ultrasound tracing of the course of the ulnar nerve may allow safe arthroscopic release (Fig. 5). The elbow should be placed in the same position (prone, elbow flexed at 90 degrees) as it will be in surgery, and the course of the ulnar nerve is identified and marked on the skin with an indelible marking pen the day before surgery. Portals are established in such way that they avoid the location of the ulnar nerve [23, 27].

#### Heterotopic ossification removal

Traditionally, removal of ectopic bone from the elbow is performed open. However, techniques have been developed for safe and effective removal of heterotopic ossification arthroscopically under selected circumstances [2]. In order to decide whether the procedure can be performed arthroscopically, it is essential to identify the relationship between areas of ectopic bone to be removed and the neurovascular structures; this is best accomplished by studying and scrutinizing preoperative computed tomography scans. If there is a reasonable distance between ectopic bone and nerves, the procedure can be performed arthroscopically. Some recommend arthroscopic removal of ectopic bone as soon as it starts to form, 2–4 weeks after the inciting injury or surgery; most recommend waiting 3 months. One benefit of very early removal is that ectopic bone is softer at that stage and may be easier to remove with just a shaver.

#### Forearm contracture release

Restoration of forearm rotation requires addressing all elements potentially involved in limiting pronosupination. Provided the only reason for limited forearm rotation is excessive scarring around the radial head and neck or a deformed radial head, arthroscopic release of scar tissue or radial head resection may allow restoration of forearm rotation arthroscopically.

#### Staging for neurogenic contractures

For patients with contractures secondary to neuritis or complex regional pain syndrome, preoperative evaluation should allow identification of the culprit nerves, typically the ulnar nerve, the posterior cutaneous nerve, or branches of the medial antebrachial cutaneous nerve. Ultrasound guided diagnostic perineural injections represent the workhorse for the evaluation of these elbows. Continued movement of the elbow after contracture release, typically recommended to maintain the motion obtained in surgery, may be ill-advised in an elbow with neuritis. As such, currently we recommend a staged approach for these patients. In a first surgery, the neuritic nerve is addressed (transposition of the ulnar nerve or transection of cutaneous nerves). Sometimes, resolution of neuritic symptoms leads to gradual recovery of motion; in many elbows, arthroscopic contracture release is required as a second stage once nerve symptoms are completely settled.

#### Stiffness in the setting of OCD

Arthroscopic management of osteochondritis dissecans is considered for those lesions that do not require cartilage replacement procedures [16]. For lesions with an intact cartilage cap, retroarticular drilling with or without bone grafting is preferred. Retroarticular drilling is performed under fluoroscopy with the C-arm in the lateral position and while visualizing the joint arthroscopically. For lesions with cartilage disruption, debridement and microfracture seem to be equivalent. Severe contractures may require a formal arthroscopic release at the time of OCD management, but most of the times, once OCD responds to treatment, contractures resolve. Anecdotical experience suggests that excessive articular arthroscopic work in patients with OCD may lead to worse arthritic changes over time.

#### Arthroscopic resection of osteoid osteomas

**Fig. 6:**
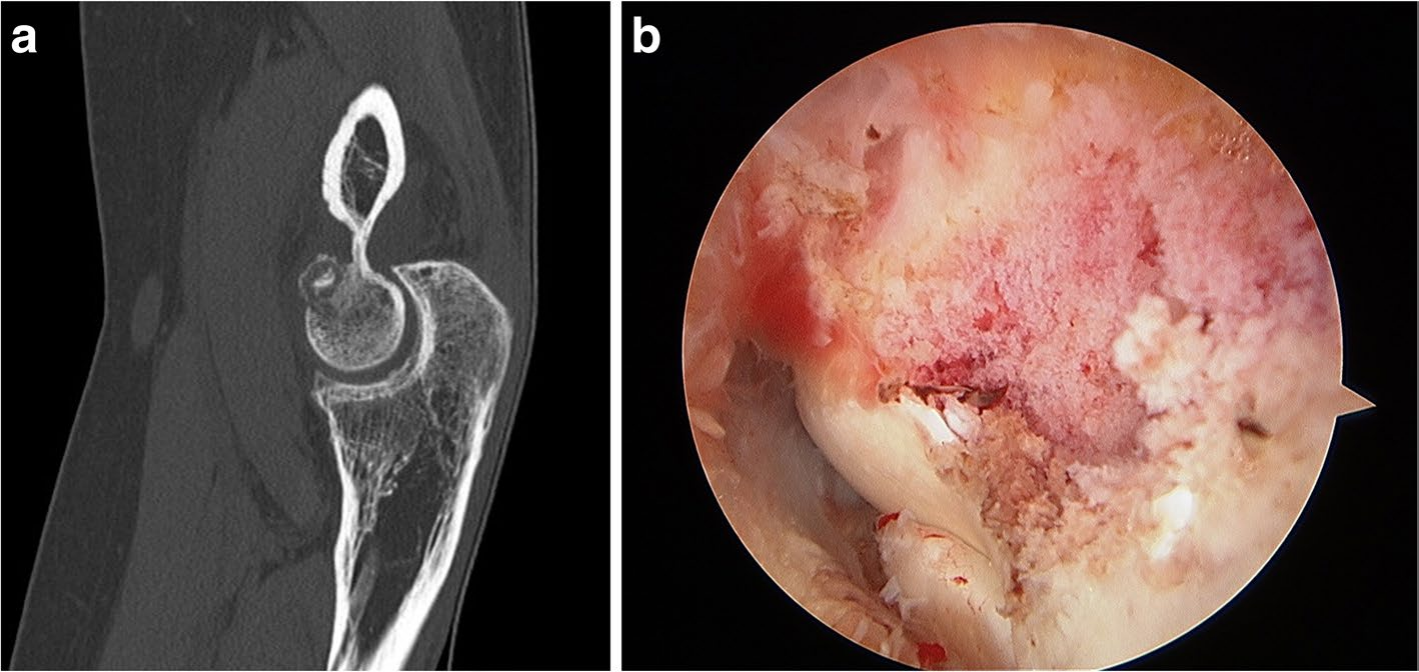
Arthroscopic removal of an osteoid osteoma. **A** Preoperative CT scan, **B** Arthroscopic view of the nidus prior to resection

Osteoid osteomas at the distal humerus, proximal ulna or radial head or neck are typically located in areas accessible to arthroscopic removal [9]. The proximity of the articular cartilage is considered a relative contraindication to radiofrequency ablation. The key for successful removal of osteoid osteomas (in addition to making the diagnosis) is to carefully analyze the three-dimensional location of the osteoma on a preoperative CT scan. Not uncommonly, the nidus is covered by sclerotic bone that must be removed before the cherry-colored tumor is visualized (Fig. 6). The osteoma should be removed with a curette or a pituitary grasper in order to be able to send to pathology a tissue sample that can be used for confirmatory diagnosis, as opposed to shaving the osteoma. Like OCD, sometimes it is not necessary to perform an arthroscopic contracture release, but moderate and severe contractures do benefit from capsulectomy.

### Postoperative management

#### Continuous passive motion

The value of continuous passive motion (CPM) has been questioned and largely discontinued after several orthopedic procedures, namely knee replacement and shoulder surgery. However, we believe that CPM does provide value for selected patients after elbow contracture release. A recently completed prospective randomized study from our Institution seems to indicate that use of CPM after arthroscopic release allows faster recovery of motion, reduces narcotic usage, and provides on average 10 more degrees of motion. The benefits of CPM are substantially larger for elbows with more severe contractures. As such, we currently recommend CPM for patients presenting with (1) severe contractures, (2) need for restoration of essentially normal motion, and (3) those interested in a faster recovery. To be effective, CPM needs to follow a specific protocol that requires use of the device most of the days and throughout the complete motion arc obtained in surgery; it typically requires temporary postoperative brachial plexus blockade to be tolerated by the patient.

#### Physical therapy

Those patients not selected for CPM treatment benefit from physical therapy exercises to reduce edema and stretch the elbow. The value of splints and braces for elbow contracture release is debated. We do consider static stretching braces for those individuals with more severe contracture who for other reasons do not undergo CPM treatment after surgery.

#### Pharmacologic adjuvants

Basic science investigations from our laboratory indicate that use of nonsteroidal anti-inflammatory drugs (in particular celecoxib) may be beneficial in the prevention and treatment of arthrofibrosis [15]. Since *indomethacin* has also been reported to decrease ectopic bone formation in other conditions, we typically recommend indomethacin for 6 weeks to all patients who undergo arthroscopic contracture release. Intraarticular *steroids* injected at the end of the procedure have been reported to be beneficial but also increase the risk of infection [20]. Some have reported anecdotical good outcomes with a short course of parenteral or oral corticosteroids. For patients with neuritis, *gabapentin* or *pregabalin* may be considered. *Antihistamines* have also been suggested to decrease arthrofibrosis experimentally, but their use in clinical practice is not completely established [31].

#### Radiation

Radiation has traditionally been considered for prevention of recurrence after ectopic bone removal [26]. Recent data from our Institution seem to indicate that radiation after open removal of ectopic bone is associated with a relatively high infection rate. Whether the same is true after arthroscopic removal remains unknown. Radiation can also be considered as a last resort for individuals presenting with refractory elbow arthrofibrosis, but again this indication is not well established.

### Complications

The most feared complications after arthroscopic contracture release of the elbow are nerve injury and infection [12, 18, 20]. Prevention of nerve injury (both nerve transection and delayed-onset ulnar neuropathy) has already been discussed [4, 10]. Other complications may include ectopic bone formation in patients without prior heterotopic ossification, aseptic fistula formation when portals are not properly closed, refracture after hardware removal when manipulation under anesthesia is performed at the end of the procedure, and cutaneous neuromas at portal sites.

### Reported outcomes

A detailed review and metanalysis of all published literature on the outcome of arthroscopic management of the stiff elbow exceeds the scope of this review article. However, review of published literature demonstrate that arthroscopic contracture release does result in improved motion for both primary and post-traumatic arthritis, and that the magnitude of motion improvement is equivalent or superior to open contracture release [14, 17, 24, 29, 33]. Although the majority of studies do not report restoration of motion to normal, most individuals included in prior studies did achieve a functional arc, and arthroscopic contracture release as also been demonstrated to restore terminal extension when needed.

### Summary

Loss of elbow motion is relatively common after trauma and can also occur as a consequence of arthritis and other conditions. Arthrofibrosis and abnormal bone impingement are present in the majority of stiff elbows. Heterotopic ossification, neurogenic contractures, osteochondritis dissecans and, rarely, osteoid osteomas may be responsible for elbow stiffness as well. History, physical examination, and radiographic findings provide useful information to make the diagnosis, to identify structures involved, and to understand motion needs. Computed tomography is the image modality of choice to complete the evaluation of most stiff elbows.

Arthroscopic contracture release, typically combined with in situ decompression of the ulnar nerve, has become the procedure of choice for the majority of elbows with primary, posttraumatic, and inflammatory arthritis. Staged surgery is commonly considered for neurogenic contractures. Surgeons with advanced arthroscopic skills may be able to tackle more complex procedures, such as arthroscopic removal of ectopic bone, release of forearm rotation contractures, and even osteoid osteoma removal. Stiffness in the context of OCD and osteoid osteomas oftentimes may not require extensive contracture release, since once the underlying pathology is addressed, milder contractures tend to resolve.

The success of arthroscopic contracture release may be largely dependent on postoperative management. Medications such as indomethacin or gabapentin/pregabalin may benefit patients with arthritis or neurogenic contractures respectively. CPM and physical therapy play an important role in the postoperative management, with CPM favored for those elbows with more severe contractures and patients that need a faster recovery or restoration of a completely normal arc of motion.

Currently, several peer-reviewed studies have reported satisfactory outcomes with arthroscopic contracture release of the elbow, equivalent or superior to open release. However, complications can happen, with infection and nerve injury as the most devastating ones. The fact that arthroscopic elbow contracture is associated with a somewhat steep learning curve cannot be overemphasized. As such, surgeons interested in these procedures should use resources available to improve their skill and always consider the complexity of each stiff elbow in light of each particular surgeon’s experience.

## References

[1] Abdel MP, Morrey ME, Barlow JD, Kreofsky CR, An KN, Steinmann SP (2012). Myofibroblast cells are preferentially expressed early in a rabbit model of joint contracture. J Orthop Res.

[2] Bachman DR, Fitzsimmons JS, O'Driscoll SW (2020). Safety of arthroscopic versus open or combined heterotopic ossification removal around the elbow. Arthroscopy.

[3] Bayram B, Owen AR, Dudakovic A, Dagneaux L, Turner TW, Bettencourt JW (2020). A potential Theragnostic regulatory Axis for Arthrofibrosis involving Adiponectin (ADIPOQ) receptor 1 and 2 (ADIPOR1 and ADIPOR2), TGFβ1, and smooth muscle α-actin (ACTA2). J Clin Med.

[4] Blonna D, Huffmann GR, O'Driscoll SW (2014). Delayed-onset ulnar neuritis after release of elbow contractures: clinical presentation, pathological findings, and treatment. Am J Sports Med.

[5] Blonna D, Lee GC, O'Driscoll SW (2010). Arthroscopic restoration of terminal elbow extension in high-level athletes. Am J Sports Med.

[6] Blonna D, O'Driscoll SW (2014). Delayed-onset ulnar neuritis after release of elbow contracture: preventive strategies derived from a study of 563 cases. Arthroscopy.

[7] Blonna D, Wolf JM, Fitzsimmons JS, O'Driscoll SW (2013). Prevention of nerve injury during arthroscopic capsulectomy of the elbow utilizing a safety-driven strategy. J Bone Joint Surg Am.

[8] Dagneaux L, Owen AR, Bettencourt JW, Barlow JD, Amadio PC, Kocher JP (2020). Human fibrosis: is there evidence for a genetic predisposition in musculoskeletal tissues?. J Arthroplast.

[9] Ge SM, Marwan Y, Abduljabbar FH, Morelli M, Turcotte RE (2020). Arthroscopic management of intra- and juxta-articular osteoid osteoma of the upper extremity: a systematic review of the literature. Eur J Orthop Surg Traumatol.

[10] Haapaniemi T, Berggren M, Adolfsson L (1999). Complete transection of the median and radial nerves during arthroscopic release of post-traumatic elbow contracture. Arthroscopy.

[11] Keener JD, Galatz LM (2011). Arthroscopic management of the stiff elbow. J Am Acad Orthop Surg.

[12] Kelly EW, Morrey BF, O'Driscoll SW (2001). Complications of elbow arthroscopy. J Bone Joint Surg Am.

[13] Keyt LK, Jensen AR, O'Driscoll SW, Sanchez-Sotelo J, Morrey ME, Camp CL (2020). Establishing the learning curve for elbow arthroscopy: surgeon and trainee perspectives on number of cases needed and optimal methods for acquiring skill. J Shoulder Elb Surg.

[14] Kwak JM, Sun Y, Kholinne E, Koh KH, Jeon IH (2019). Surgical outcomes for post-traumatic stiffness after elbow fracture: comparison between open and arthroscopic procedures for intra- and extra-articular elbow fractures. J Shoulder Elb Surg.

[15] Limberg AK, Tibbo ME, Salib CG, McLaury AR, Turner TW, Berry CE (2020). Reduction of arthrofibrosis utilizing a collagen membrane drug-eluting scaffold with celecoxib and subcutaneous injections with ketotifen. J Orthop Res.

[16] Logli AL, Bernard CD, O'Driscoll SW, Sanchez-Sotelo J, Morrey ME, Krych AJ (2019). Osteochondritis dissecans lesions of the capitellum in overhead athletes: a review of current evidence and proposed treatment algorithm. Curr Rev Musculoskelet Med.

[17] Lubiatowski P, Ślęzak M, Wałecka J, Bręborowicz M, Romanowski L (2018). Prospective outcome assessment of arthroscopic arthrolysis for traumatic and degenerative elbow contracture. J Shoulder Elb Surg.

[18] Marti D, Spross C, Jost B (2013). The first 100 elbow arthroscopies of one surgeon: analysis of complications. J Shoulder Elb Surg.

[19] Martinez-Catalan N, Sanchez-Sotelo J (2021). Primary elbow osteoarthritis: evaluation and management. J Clin Orthop Trauma.

[20] Nelson GN, Wu T, Galatz LM, Yamaguchi K, Keener JD (2014). Elbow arthroscopy: early complications and associated risk factors. J Shoulder Elb Surg.

[21] Nesterenko S, Sanchez-Sotelo J, Morrey BF (2009). Refractory elbow arthrofibrosis. A report of four cases. J Bone Joint Surg Am.

[22] O'Driscoll SW (1995). Arthroscopic treatment for osteoarthritis of the elbow. Orthop Clin North Am.

[23] Park SE, Bachman DR, O'Driscoll SW (2016). The safety of using proximal Anteromedial portals in elbow arthroscopy with prior ulnar nerve transposition. Arthroscopy.

[24] Rai S, Zhang Q, Tamang N, Jin S, Wang H, Meng C (2019). Arthroscopic arthrolysis of posttraumatic and non-traumatic elbow stiffness offers comparable clinical outcomes. BMC Musculoskelet Disord.

[25] Ranganathan K, Loder S, Agarwal S, Wong VW, Forsberg J, Davis TA (2015). Heterotopic ossification: basic-science principles and clinical correlates. J Bone Joint Surg Am.

[26] Robinson CG, Polster JM, Reddy CA, Lyons JA, Evans PJ, Lawton JN (2010). Postoperative single-fraction radiation for prevention of heterotopic ossification of the elbow. Int J Radiat Oncol Biol Phys.

[27] Sahajpal DT, Blonna D, O'Driscoll SW (2010). Anteromedial elbow arthroscopy portals in patients with prior ulnar nerve transposition or subluxation. Arthroscopy.

[28] Sanchez-Sotelo J (2016). Elbow rheumatoid elbow: surgical treatment options. Curr Rev Musculoskelet Med.

[29] Schreiner AJ, Schweikardt N, Gühring D, Ahrend MD, Döbele S, Ahmad SS (2020). Arthroscopic arthrolysis leads to improved range of motion and health-related quality of life in post-traumatic elbow stiffness. J Shoulder Elb Surg.

[30] Sears BW, Puskas GJ, Morrey ME, Sanchez-Sotelo J, Morrey BF (2012). Posttraumatic elbow arthritis in the young adult: evaluation and management. J Am Acad Orthop Surg.

[31] Tibbo ME, Limberg AK, Salib CG, Turner TW, McLaury AR, Jay AG (2020). Anti-fibrotic effects of the antihistamine ketotifen in a rabbit model of arthrofibrosis. Bone Joint Res.

[32] Williams BG, Sotereanos DG, Baratz ME, Jarrett CD, Venouziou AI, Miller MC (2012). The contracted elbow: is ulnar nerve release necessary?. J Shoulder Elb Surg.

[33] Willinger L, Siebenlist S, Lenich A, Liska F, Imhoff AB, Achtnich A (2018). Arthroscopic arthrolysis provides good clinical outcome in post-traumatic and degenerative elbow stiffness. Knee Surg Sports Traumatol Arthrosc.

